# Impact of External Mechanical Loads on Coda Waves in Concrete

**DOI:** 10.3390/ma15165482

**Published:** 2022-08-09

**Authors:** Fabian Diewald, Niklas Epple, Thomas Kraenkel, Christoph Gehlen, Ernst Niederleithinger

**Affiliations:** 1 Centre for Building Materials, Technical University of Munich, 81245 Munich, Germany; 2 Bundesanstalt für Materialforschung und-Prüfung, 12205 Berlin, Germany

**Keywords:** ultrasound, coda wave interferometry, concrete, mechanical load, microstructure, monitoring, non-destructive testing, civil engineering

## Abstract

During their life span, concrete structures interact with many kinds of external mechanical loads. Most of these loads are considered in advance and result in reversible deformations. Nevertheless, some of the loads cause irreversible, sometimes unnoticed changes below the macroscopic scale depending on the type and dimension of the impact. As the functionality of concrete structures is often relevant to safety and society, their condition must be known and, therefore, assessed on a regular basis. Out of the spectrum of non-destructive monitoring methods, Coda Wave Interferometry using embedded ultrasonic sensors is one particularly sensitive technique to evaluate changes to heterogeneous media. However, there are various influences on Coda waves in concrete, and the interpretation of their superimposed effect is ambiguous. In this study, we quantify the relations of uniaxial compression and uniaxial tension on Coda waves propagating in normal concrete. We found that both the signal correlation of ultrasonic signals as well as their velocity variation directly reflect the stress change in concrete structures in a laboratory environment. For the linear elastic range up to 30% of the strength, we calculated a velocity variation of −0.97‰/MPa for compression and 0.33%/MPa for tension using linear regression. In addition, these parameters revealed even weak irreversible changes after removal of the load. Furthermore, we show the time-dependent effects of shrinkage and creep on Coda waves by providing the development of the signal parameters over time during half a year together with creep recovery. Our observations showed that time-dependent material changes must be taken into account for any comparison of ultrasonic signals that are far apart in time. The study’s results demonstrate how Coda Wave Interferometry is capable of monitoring stress changes and detecting even small-size microstructural changes. By indicating the stated relations and their separation from further impacts, e.g., temperature and moisture, we anticipate our study to contribute to the qualification of Coda Wave Interferometry for its application as an early-warning system for concrete structures.

## 1. Introduction

Reinforced concrete is used for a large part of our built environment today [1], especially in engineering structures such as our infrastructure. Besides the essential safety aspect, reliability and permanent availability are major objectives regarding these structures’ design and maintenance. Higher demands, especially due to rising traffic volume and heavy load traffic, and the simultaneous aging of the structures, necessitate increased attention to maintenance and monitoring to avoid disruptive effects such as local material failure or even a devastating collapse.

Regular inspections, in most cases based on visual examination or tap tests, ensure structural integrity and operability. On the downside, they are only able to represent the structure’s state at accessible areas at one point in time, whereas monitoring methods, usually from the field of non-destructive testing (NDT), complement the spectrum of available tools for safety assurance. Reinforced concrete, however, poses a special challenge for NDT techniques due to the material’s heterogeneity, which complicates the separation between naturally present scatterers and undesired material changes, or damage. To this end, the evaluation of ultrasonic signals by means of Coda Wave Interferometry (CWI) is a promising and exceptionally sensitive method to detect even weak changes in the material and has been subject to previous studies on both the laboratory [2,3,4,5,6], and the structural scale [7,8,9,10]. It is suitable for application as a permanent monitoring system with the focus on the early warning of microcrack initiation, and possibly providing the impulse for further in-depth inspections at alarming locations.

With this study, we quantify the effects of uniaxial compression, uniaxial tension, shrinkage and creep of a normal concrete on signal changes in the Coda that we describe by means of correlation coefficients and velocity variations of collected ultrasonic signals.

## 2. State of the Art

### 2.1. Coda Wave Interferometry

**Figure 1:**
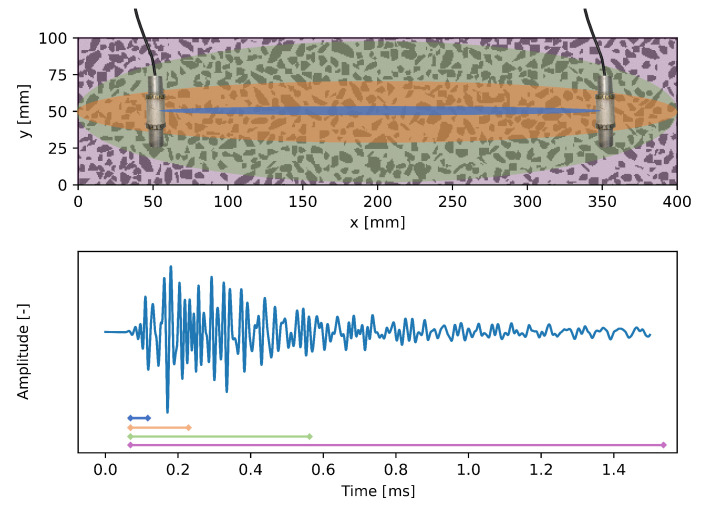
Ultrasonic recording (**bottom**) and the schematic explanation of CWI with two US transducers in a concrete model (**top**): when analyzing only the first arriving waves, the measurements are sensitive only to the area around the direct path between source and receiver (blue). As more scattered waves are included, the area increases (orange, green). Referring to the entire signal (purple), scattered waves from the entire medium are recorded at the receiver.

Ultrasonic (US) waves in inhomogeneous media are scattered, depending on the wave properties such as frequency, and the size, shape, and type of the inhomogeneities. The effect of such scattering on ultrasonic measurements is shown in Figure 1. Although we record a distinct first arrival, which is used in classic US non-destructive testing (NDT) methods, a significant portion of the energy emitted at the source arrives at the receiver later than this first arrival. This long tail of the measurement is called Coda. Although the origin of Coda wave-based methods is found in the field of seismology, the analysis of such waves by Coda Wave Interferometry has been applied to monitoring of solids in recent decades (see e.g., [11,12,13,14]). Using CWI methods, evaluation of consecutive ultrasound measurements enables detecting weak changes to the signal. This technique is based on the fact that the medium is acting as an interferometer, merging the scattered waves in the receiver position [12]. As long as the medium (interferometer) does not change, repeated measurements will record identical signals. Changes to the medium as a result of local or global impacts directly affect the recorded signals. If the change is between source and receiver, it will be visible in the first arrival. Changes outside the direct path are only affecting the scattered waves and can, therefore, only be detected using CWI analysis of volumetric ultrasound information (Figure 1).

Generally, CWI methods take advantage of the accumulation of weak changes over a great number of wave paths between a sender-receiver pair that are caused by the heterogeneities. Multiple methods for CWI analysis have been proposed in recent decades [12,14,15], all based on the calculation of the signal correlation coefficient (CC). This coefficient determines the similarity of a reference signal u(t) in comparison to a signal recorded after a perturbation in the material $\tilde{u}\left(t\right)$. In this work, we will use the stretching technique (see [15]) to determine apparent relative velocity changes (dv/v) in the medium. This method applies different stretching factors ε=dv/v to the reference signal and determines the ε maximizing CC:

(1) $$CC\left(\varepsilon \right)=\frac{\int_{t_{1}}^{t_{2}}u\left(t\right)\tilde{u}\left(t\left(1-\varepsilon \right)\right)\;dt}{\sqrt{\int_{t_{1}}^{t_{2}}u^{2}\left(t\right)\;dt\int_{t_{1}}^{t_{2}}{\tilde{u}}^{2}\left(t\left(1-\varepsilon \right)\right)\;dt}}$$

With this method, two parameters, ε=dv/v and CC can be extracted from the ultrasonic coda in the time interval $\left[t_{1},t_{2}\right]$ and be used to analyze and track global and local material changes. The reference measurement u(t) can either be fixed for an entire experiment or change during the course of it if the differences between u(t) and $\tilde{u}\left(t\right)$ become too large (e.g., CC≤0.7) due to damage or material property changes [16]. Both the fixed reference and the changing reference method can be applied to relate the velocity change in a monitoring experiment to the initial state of the specimen. Although for the fixed reference method this is straightforward, a stepwise change in reference requires cumulative calculation of the total velocity change at every step. When switching from fixed reference to the stepwise method, CC can no longer be used for interpretation.

### 2.2. Mechanical Loading of Concrete

Concrete is a complex heterogeneous material consisting of coarse aggregates and a system of air pores embedded into a cementitious mortar matrix. Depending on the design case and objective, concrete can be investigated on different scales, whereas most structural and material design models consider the material on the macro level as a homogeneous material. However, evaluation of crack formation starts with pre-existing defects on the micro level, followed by a multilayered process of damage evolution [17].

**Figure 2:**
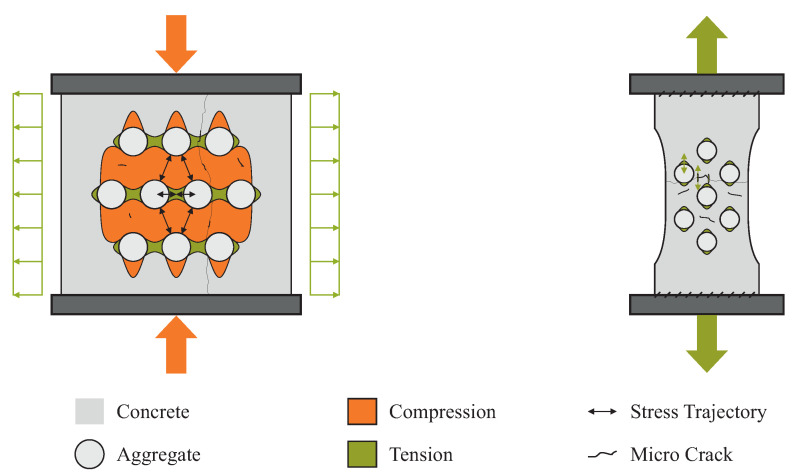
Uniaxial loading of normal concrete specimens, where the cement paste’s Young’s modulus is lower in comparison to the aggregate’s Young’s modulus. The experimental setups for both compression (**left**), and tension (**right**), correspond to the experiments in Section 3. The compression case is shown with the assumption of no friction at the contact surface between press and specimen.

From the monitoring perspective, it is most ideal to detect weak changes in the material already on the microstructural level and use the collected information as a precursor for material failure on the above-lying scales. Besides the impact of environmental conditions such as temperature or moisture, changes are in many cases caused by mechanical loads, i.e., compression, tension, or multidimensional stress states. We show the crack formation process for a normal concrete for the uniaxial compression and tension cases in Figure 2, which we cover in our experiments.

Pre-existent micro-cracks evolve primarily in tensile sections along the comparably weaker boundary between the cement matrix and single aggregates, the Interfacial Transition Zone [18]. For the compression case with much higher strength, stress trajectories appear between adjacent aggregates [19], whereas for tension, a single separating crack develops [20].

Besides direct impacts such as short-term loading, time-dependent phenomena such as shrinkage and creep are characteristic for concrete. Shrinkage causes a reduction in the material’s volume and is affected by the ambient humidity, the component geometry, the drying process, and the concrete mix. It is a superimposed process of drying shrinkage due to moisture loss to the environment and autogenous shrinkage due to the internal reaction of water during the hydration from fresh to hardened concrete [21]. Although deformations as a consequence of shrinkage are generally load-independent, creep deformations are considered to depend primarily on the load level and its duration of exposition but also on moisture migration and micro crack formation [22].

**Figure 3:**
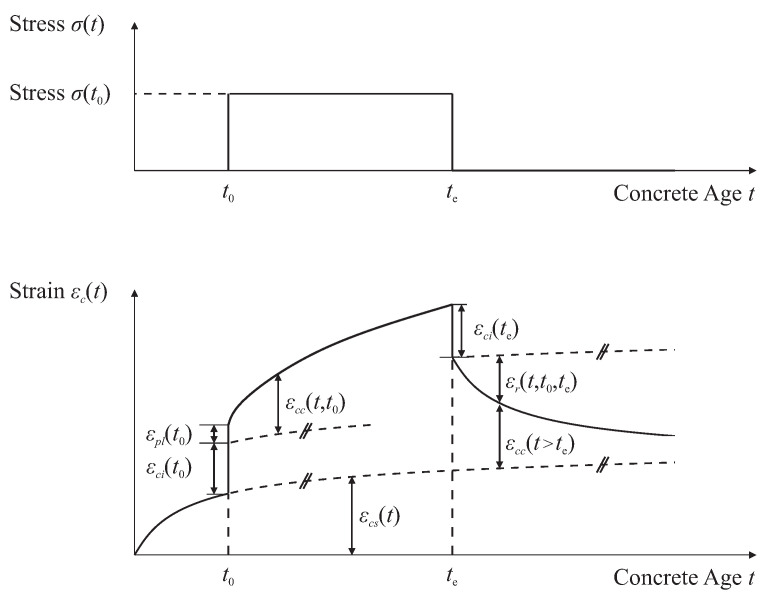
Stress (**top**) and corresponding strain (**bottom**) for shrinkage and creep in concrete as a function of concrete age *t*. The characteristic points $t_{0}$ and $t_{e}$ specify loading and unloading with a constant load $\sigma (t_{0})$.

The immediate strain $\varepsilon_{ci}\left(t_{0}\right)$, together with a plastic term $\varepsilon_{pl}\left(t_{0}\right)$, is complemented by two terms that account for shrinkage $\varepsilon_{cs}\left(t_{s}\right)$ and creep $\varepsilon_{cc}\left(t\right)$ for a constant compressive stress $\sigma (t_{0})$ during the loading period between $t_{0}$ and $t_{e}$. Equation (2) [23] summarizes the total strain $\varepsilon_{c}\left(t\right)$ (Figure 3) for a load application at $t_{0}$ at constant environmental conditions.

(2) $$\varepsilon_{c}\left(t\right)=\varepsilon_{cs}\left(t\right)+\varepsilon_{ci}\left(t_{0}\right)+\varepsilon_{pl}\left(t_{0}\right)+\varepsilon_{cc}\left(t,t_{0}\right)$$

## 3. Materials and Methods

### 3.1. Experimental Setup

We used an identical concrete mix for all experiments. Table 1 shows the concrete raw materials and their composition. Pre-tests for the Young’s modulus yielded 84.6 GPa for the mainly quartzitic crushed aggregates and 27.1 GPa for the hardened mortar. The Poisson’s Ratio was 0.12 for the aggregates and 0.19 for the mortar. The compressive strength was 368.0 MPa for the aggregates and 80.3 MPa for the mortar. A standardized pressure method test yielded an air void content of 3.9% in the fresh concrete. The polycarboxylatether-based superplasticizer was added to the mixing water to improve the concrete’s workability and reduce the cement paste viscosity to enhance the sensor-to-concrete coupling condition. The characteristic concrete parameters were additionally modeled [23], particularly considering the concrete model’s damage evolution [24]. After production, the specimens were cured at 20 °C under water for 7 days and restored to 65% RH and 20 °C. We started each experiment in this study on the 28th day after concrete production.

### 3.2. Compression Experiment

**Figure 4:**
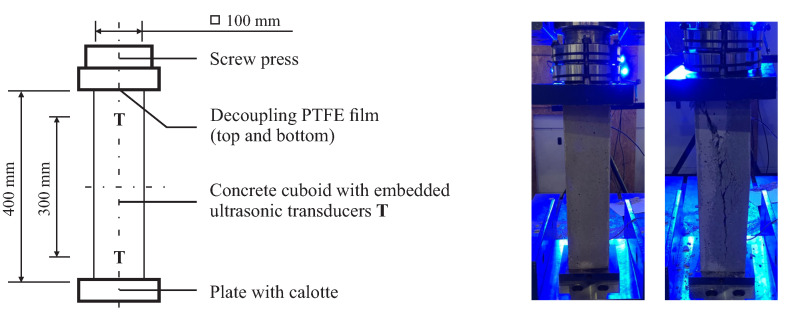
Setup for the uniaxial compression experiment: (**left**) sketch of the cuboid concrete specimens with their dimensions, (**center**) picture of the experiment, and (**right**) picture after failure. A pre-test with three identical specimens yielded a compressive strength of 42.9 MPa.

For the compression test, we used three cuboid specimens with an edge length of 400 mm and side lengths of 100 mm with a transducer distance of 300 mm symmetrical to the center axis (Figure 4). We decoupled the specimen on the sawn end faces by a polytetrafluorethylen (PTFE) film from the hydraulic press to minimize friction, thus avoiding a multiaxial stress state next to the faces. In advance, we evaluated the average compressive strength $f_{c}$ of three geometrically identical samples without embedded transducers, which was 42.9 MPa. Subsequently, we applied a compressive load to the actual specimens with a loading rate of 50 N/s while recording ultrasonic signals between the embedded transducers every 10 s. In advance, all specimens were loaded at 5% $f_{c}$ to dissolve initial internal stress. We defined different maximum loads for each specimen, i.e., 100%, 60%, and 30% of the compressive strength $f_{c}$. Having reached the maximum load, we unloaded the sound specimens again.

### 3.3. Tension Experiment

**Figure 5:**
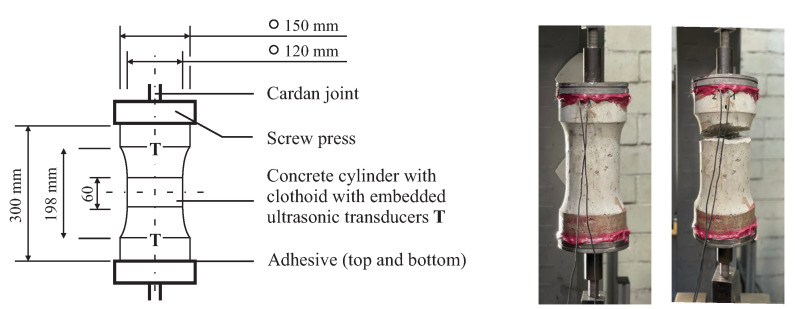
Setup for the uniaxial tension experiment: (**left**) sketch of the cylindrical concrete specimens with their dimensions, (**center**) picture during the experiment, and (**right**) picture after failure. A pre-test with three identical specimens yielded a tensile strength of 3.0 MPa.

For the centric tension test, we used three cylindrical bone-shaped specimens with a length of 300 mm and a decreasing cross section [20] to define a predetermined fracture section (Figure 5). The transducer distance was 198 mm symmetrical to the center axis. The specimens were fastened at the tensile machine using the two-component adhesive *Hilti HIT-RE 500 V3* having cut the end faces in a chessboard pattern to improve the strength of the joint. The tensile strength was determined to be 3.0 MPA beforehand from three identical samples. The subsequent loading rate was 5 N/s while recording every 10 s up to maximum loads of 100%, 60%, and 30% of the tensile strength $f_{ctm}$. Like the compression test, we unloaded the sound specimens again.

### 3.4. Shrinkage and Creep Experiment

**Figure 6:**
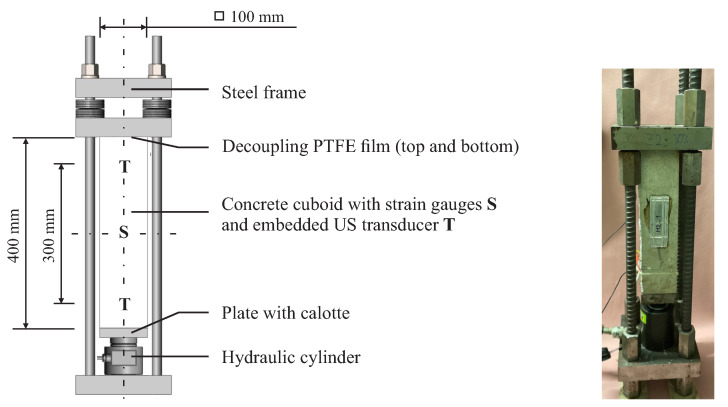
Setup for the shrinkage and creep experiment: (**left**) sketch of the cuboid concrete specimens with their dimensions, loaded within a steel frame, and (**right**) picture of the experiment.

The specimens for the shrinkage and creep tests were cuboids, geometrically equal to the ones from the compression test. The edge length was 400 mm and side lengths were 100 mm with a transducer distance of 300 mm symmetrical to the specimen’s center axis (Figure 6). The sawn end faces were decoupled by a PTFE film from the steel frames in which a hydraulic system instantaneously compressed the specimens at different constant loads of 11%, 18%, and 33% of the previously determined compressive strength. In addition, we investigated a sealed specimen at a load of 33% and an unloaded specimen to monitor pure shrinkage. We applied the load for the duration of half a year and continued monitoring for another 50 days after unloading to observe creep recovery.

The normal strain of every specimen was individually tracked by a system of strain gauges in a temperature-compensated circuit of two active and two passive strain gauges each. All specimens were equipped with embedded piezoelectric transducers (described in [25]) to collect ultrasonic signals. The transducers acted as sender-receiver pairs with a center frequency of 60 kHz. The measurement system (described in [26]) recorded the resulting signal with a sampling rate of 1 MHz and 12,000 samples with a resolution of 14 bits. We subsequently compared the signals without further averaging.

## 4. Results

For each experiment, we evaluated the collected ultrasonic signals using Coda Wave Interferometry methods. By comparing every signal to a fixed, non-perturbed reference signal at an unloaded state, we present the velocity variations dv/v together with the signal correlation coefficient CC according to Equation (1) during the individual loading scenarios. Positive values of dv/v indicate a velocity decrease and vice versa. All ultrasound measurements were preprocessed before application of the CWI algorithm. Preprocessing included offset compensation, pretrigger and crosstalk removal and frequency filtering. We refer to [16] for the detailed processing procedure. CWI parameter changes as a consequence of either compressive or tensile stress may be interpreted as calibration curves, whereas the development of CWI parameters over time due to the phenomena shrinkage and creep are referred to as concrete-specific features.

### 4.1. Uniaxial Compression

**Figure 7:**
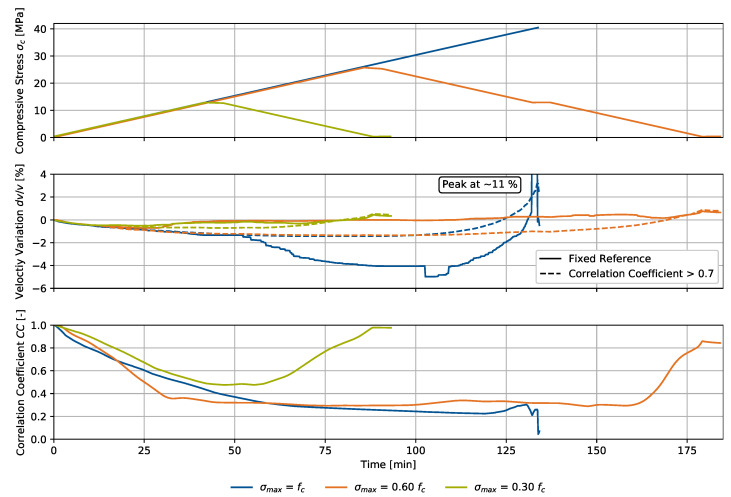
Uniaxial compression experiment for three specimens, referring to the setup in Figure 4 with different maximum stresses: compressive stress $\sigma_{c}$, velocity variation dv/v and signal correlation coefficient CC over time.

The compressive loading scenario is presented in Figure 7 together with the velocity variation dv/v and the signal correlation coefficient CC for a fixed reference signal in an unloaded state. The loading and unloading rate was 50 N/s and equal for all specimens. One specimen was loaded up to failure ($\sigma_{max}=f_{c}$), which caused maximum velocity variation at failure of approximately 11%. The other specimens were loaded up to 30 and 60% of the maximum load $f_{c}$. The maximum load was held for 5 min, followed by unloading. During the whole experiment, we left the press connected to the specimen by applying a minimum load of 1% $f_{c}$ to keep the contact condition of the press unchanged. Ultrasonic signals were collected at a rate of 0.1 Hz.

As the experiment covers not only weak changes in the stress state, using one fixed reference for the entire experiment may cause discontinuities of velocity perturbations due to great signal variations in comparison to an unloaded state. We considered this method-specific phenomenon by establishing an additional evaluation technique picking a new reference signal each time the overall signal correlation coefficient drops below a threshold, which was defined as 0.7 for this analysis. Therefore, we ensure keeping the signal changes within the thus generated windows small enough to satisfy the principles of Coda Wave Interferometry regarding the sensitive evaluation of weak changes to the material. Up to a compressive stress of 6.4 MPa, equaling $0.15f_{c}$, we observe insignificant deviations between the two methods for the development of dv/v. For a growing stress, deviations increasingly change and the gradient switches sign for the two specimens $\sigma_{max}=0.30f_{c}$ and $\sigma_{max}=0.60f_{c}$. Numerical instabilities are observed particularly for the specimen $\sigma_{max}=f_{c}$.

Analysis of the velocity variation yielded similar development over time, or equally load. We observed a steady signal compression for increasing loads up to approximately 25.6 MPa, equaling 60% $f_{c}$, resulting in a maximum dv/v of −1.6%. For further loading, the velocity variation gradient switched sign and increasingly rose, indicating evolving damage in the material. For the two non-failed specimens, dv/v turned positive while unloading and reached a maximum of 0.6% for $\sigma_{max}=0.30f_{c}$ and 0.9% $\sigma_{max}=0.60f_{c}$, indicating irreversible material changes after the experiment. A linear regression analysis of the three specimens up to $0.30f_{c}$ yielded a velocity variation increase of −0.97 ‰/MPa which is comparable to the slope in [27]. The coefficient of determination for this analysis is 76.6%.

Analysis of the signal correlation coefficient development also yielded similar results for all specimens up to 12.8 MPa, equaling 30% $f_{c}$, where CC expectedly drops for increasing loads. For CC below 0.4 to 0.3, its gradient is attenuated, and does not drop below 0.2 before material failure. Regarding the two unloaded specimens, we observed an increase in CC, but not a complete restoration of the original signal at the unloaded state. The remaining offset was approximately 0.03 for $\sigma_{max}=0.30f_{c}$ and 0.15 for $\sigma_{max}=0.60f_{c}$. As environmental and bearing conditions did not change during the experiment, the remaining offset is connected to the internal material damage of the specimen, which is significantly larger for higher loads, and corresponds to the observations referring to the signals’ velocity variation dv/v.

**Figure 8:**
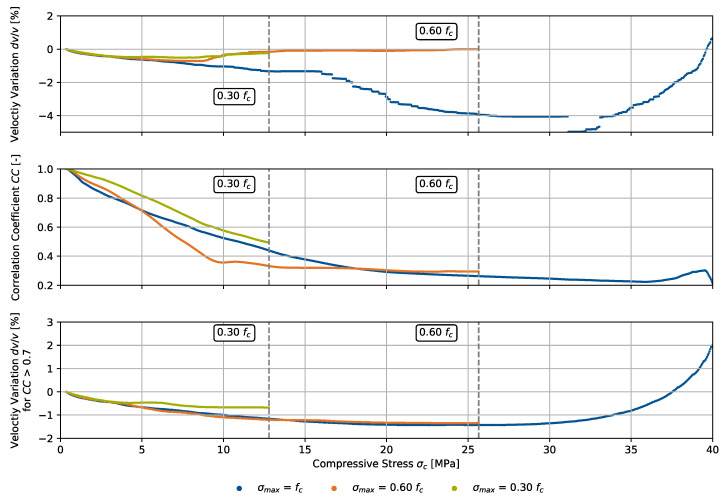
Velocity variation dv/v and signal correlation coefficient CC for three specimens as in Figure 7, translated into the compressive stress domain during the loading process up to 100% (failure), 60%, and 30% of the compressive strength $f_{c}$.

Both parameters were translated into the compressive stress domain during the loading process. We show the results in Figure 8.

The velocity variation is plotted in accordance with both methods for one fixed reference (top) and switching reference signals for windows with CC > 0.7 (bottom). As both ultrasonic signals and the stress have been measured in the time domain, CWI parameters are linearly interpolated for the respective stress. For a measuring rate of the load of 10 Hz and a loading rate of 50 N/s, we specify a resolution of < 0.25 MPa. Evaluating CC only above the threshold 0.7 yields a steady development of dv/v for increasing loads up to $\sigma_{max}=0.60f_{c}$, whereas dv/v is not completely steady, in particular for sections with major changes to the ultrasonic signals.

Considering building codes [28], the elastic modulus of concrete can be estimated referring to its strength, depending on the used aggregates. For the concrete with quartzitic aggregates, the secant modulus between $\sigma_{c}=0$ and $40\%f_{c}m$ is applied for determining the elastic modulus, where $f_{c}m$ is the medium compressive strength. Referring to the standards, the relation between compressive stress and the CWI parameters is nearly reversible within this range, which has been confirmed up to 30% $f_{c}$ in this experiment through an approximate restoration of the original values of dv/v and CC after unloading. However, deviations as a consequence of damage evolution on a microstructural level must be expected due to the heterogeneous nature of concrete.

### 4.2. Uniaxial Tension

**Figure 9:**
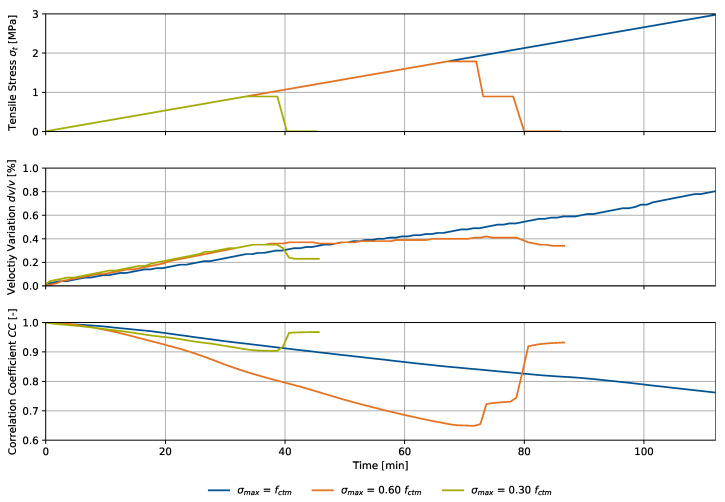
Uniaxial tension experiment for three specimens, referring to the setup in Figure 5 with different maximum stresses: tensile stress $\sigma_{t}$, velocity variation dv/v and signal correlation coefficient CC over time.

The centric tensile loading scenario is presented in Figure 9 together with the velocity variation dv/v and the signal correlation coefficient CC. The indicated tensile stress refers to the center circular cross section with a diameter of 120 mm. The loading rate was 5 N/s and equal for all specimens. As in the compression experiment, one specimen was loaded up to failure, whereas two specimens were loaded to 30% and 60% of the maximum load $f_{ctm}$. The maximum load was held for 5 min, followed by unloading. The contact condition during the experiment stayed unchanged due to the adhesive connection. Therefore, no minimum load, as during the compression experiment, was applied. Ultrasonic signals were collected at a rate of 0.1 Hz.

In comparison to the compressive loading experiment, the stress range is smaller due to the characteristic behavior of concrete regarding tension. For this concrete mix, the ratio between the tensile strength $f_{ctm}$ and the compressive strength was determined to be around 7.0%. We show dv/v for a fixed reference signal in an unloaded state only, since CC does not drop as significantly as during compression. Therefore, we consider one fixed reference sufficient to cover the entire experiment.

Analysis of the velocity variation yielded similar forms for all specimens over time, or equally load. Signals were steadily stretched for increasing tensile loads up to 100% fctm, or 3.0 MPa. We observed nearly linear development of dv/v, whereas the gradient of one specimen with a maximum load of $\sigma_{max}=0.60f_{ctm}$ rapidly decreased above approximately 30% $f_{ctm}$. The maximum velocity variation before failure was 0.80%. Failure was characterized by evolution of one characteristic macro crack. Having reached their respective maxima at 0.36% for $\sigma_{max}=0.30f_{ctm}$ and 0.41% for $\sigma_{max}=0.60f_{ctm}$, dv/v decreased while unloading the specimens. As in the compression experiments, the remaining velocity variation was higher for greater maximum loads, 0.22% for $\sigma_{max}=0.30f_{ctm}$ and 0.37% $\sigma_{max}=0.60f_{ctm}$, again indicating irreversible material changes after the experiment. A linear regression analysis of the three specimens up to $0.30f_{ctm}$ yielded a velocity variation increase of 0.33%/MPa. The coefficient of determination for this analysis is 92.2%.

Signal correlation coefficients steadily decreased during the experiment time, or load up to $f_{ctm}$, whereas deviations between the specimens’ CCs also increased for growing tensile stress. The minimum CCs were computed at 0.90 for $\sigma_{max}=0.30f_{ctm}$ and 0.66 for $\sigma_{max}=0.60f_{ctm}$. After unloading, offsets remained in both cases despite unchanged environmental and bearing conditions. The individual offsets depended on the applied maximum load according to the observations for dv/v. For greater loads, the offset increased, which related to irreversible material changes on a microstructural level.

**Figure 10:**
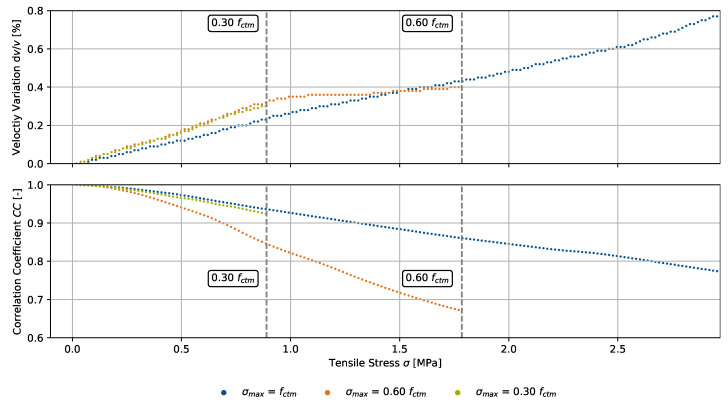
Velocity variation dv/v and signal correlation coefficient CC, both using a fixed reference signal at an unloaded state, for three specimens as in Figure 9, translated into the tensile stress domain during the loading process up to 100% (failure), 60%, and 30% of the compressive strength $f_{ctm}$.

We translated both CWI parameters into the tensile stress domain during the loading process and show the results for the three specimens in Figure 10. Likewise, the compression experiment, CWI parameters are linearly interpolated for the respective stress. For a measuring rate of the load of 10 Hz and a loading rate of 5 N/s, we specify a resolution of <0.025 MPa. Since the parameters showed a steady increase for greater loads at a constant loading rate, their developments in the tensile stress domain are equivalent to the time domain.

### 4.3. Shrinkage and Creep

**Figure 11:**
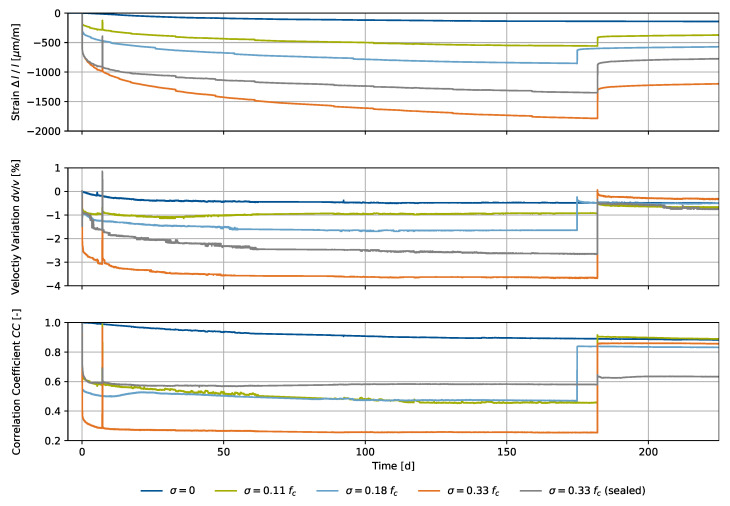
Shrinkage and creep experiment with different constant stresses referring to the setup in Figure 6: strains Δl/l, velocity variations dv/v and signal correlation coefficient CC over time.

Both processes, shrinkage and creep, are concrete-specific phenomena. In contrast to external load variations, which have a direct impact on the ultrasonic signal, these phenomena cause a characteristic material reaction over time. In this experiment, we show the CWI parameters, together with the measured strain, for pure shrinkage and superimposed creep at different constant loads of $\sigma =\left[0.11\%,18\%,33\%\right]\;f_{c}$ in Figure 11.

The specimens were loaded for the duration of half a year and unloaded after that period. The spike after 7 days results from a restart of the load-applying hydraulic system, where further specimens were added to the experiment. Therefore, the experiment duration for $\sigma =18\%\;f_{c}$ is 7 days shorter in comparison to the remaining specimens. Ultrasonic signals were collected approximately every 10 min using a *Raspberry Pi*-based measuring system [26]. CWI parameters result from evaluation using a fixed reference, immediately after the initial load application. The environmental conditions were 20 °C and 65% RH. Like the bearing conditions, they stayed unchanged during the entire experiment.

The CWI analysis by means of a fixed reference signal, immediately after load application, showed a signal velocity variation corresponding to the characteristic strain evolution during concrete shrinkage and creep. For pure shrinkage, we observed −0.4% dv/v after half a year, whereas the specimens exposed to external loads showed values up to −3.8% dv/v for the maximum load. The resulting values of dv/v sequence according to the amount of the applied load. After unloading, all values of dv/v spontaneously returned towards their original values. As all specimens are simultaneously exposed also to shrinkage, the remaining strains cause the CWI parameters not to return completely to zero. However, there are additional changes to the material, regarding strain after unloading. Furthermore, creep recovery of the material appears, also detectable in both strain and velocity variation.

Signal correlation coefficients CC significantly decrease instantly after load application as a consequence of large deformations. They are considered ambiguous, especially for small values of CC, i.e., for most creep scenarios. For pure shrinkage, CC remained above 0.85 during the entire experiment, where its development reflects the typical shrinkage behavior, validated by this specimen’s strain.

## 5. Discussion

The propagation of Coda waves in concrete is affected by various external impacts that may cause microstructural changes to the material. To interpret the total signal changes, the single effects must be separable, particularly regarding reversible and irreversible changes, or damage. By means of our experiments, we can specify the relations between externally applied mechanical loads, i.e., uniaxial compression and tension, and immediate changes to the ultrasonic signals, i.e., signal correlation and velocity variation, using Coda Wave Interferometry methods. We indicate these relations as calibration curves for a normal concrete, whereas we assess a full range of such calibration curves as key to separate reversible from irreversible changes. This range must be extended by further impacts, i.e., temperature and moisture for the identical material in the future.

As we produced a standard concrete using Ordinary Portland Cement, quartzitic aggregates, and a w/c-ratio of 0.45, we evaluate the relations as references valid for a broad range of concrete mixes. However, we expect deviations for concrete mixes with varying cement stone and aggregate stiffness due to the modified crack formation process. During the compression experiment, we consider CWI evaluation techniques with a single fixed reference not able to cover the entire stress range between the unloaded state and material failure due to major material changes, especially while irreversible damage advance. The evolution of dv/v can therefore be ambiguous due to abrupt changes to the maximized signal correlation CC(t,ε), but without a significant physical material change. A fixed reference signal in the unloaded state has proven to be insufficient to detect advancing damage. Therefore, we propose the use of CWI evaluation for a range, where only moderate material changes are expected. We address this hypothesis by the introduction of a tailored method, which switches the reference signal every time the signal correlation drops between a fixed value of 0.7. We recognize further optimization potential regarding this condition to cover small- and large-scale material changes at the same time using condition-based signal reference.

Furthermore, we stated a calibration curve for the impact of uniaxial tension on the same concrete mix using one fixed reference signal at the unloaded state. In this experiment, we found nearly linear relations of dv/v and CC for increasing stress as a consequence of a purely uniaxial stress state. Thus, linear crack evolution of single cracks was observed up to failure, completed by the specimens’ fracture forming one single macro crack. For both experiments, we showed irreversible changes in the velocity variation dv/v by a remaining velocity decrease for both compression and tension, relating to irreversible micro-cracks in the material.

In addition to immediate responses of the CWI parameters, we extended our experiments by investigation of concrete-characteristic shrinkage and creep behavior. These phenomena were monitored in the time domain and are, in contrast to calibration curves, considered to be long-term features. We showed that shrinkage and creep behavior is, in addition to conventional techniques such as strain measurement, representable by evaluation of the velocity variation dv/v. By implication for long-term monitoring processes, it is pivotal to compensate for concrete-typical effects when interpreting two far apart states in time. These time-dependent changes must be considered for signal comparisons by means of calibration curves as the reference may have changed, even if the impacts of stress, temperature and moisture are identical. Further phenomena such as carbonation, freeze attack, or steel corrosion in reinforced concrete will have to be accounted for beyond our investigations.

## 6. Conclusions

Coda Wave Interferometry is a superiorly sensitive method to detect weak changes to heterogeneous materials such as concrete. To establish this technology as an early-warning system for real structures, we analyzed the relations between the correlations and velocity variations of the ultrasonic signals using the stretching technique and externally applied mechanical loads, which we call calibration curves. These curves could serve as the basis for translation of multidimensional fields of velocity variations into stress changes and damage, even for more-than-one-dimensional stress states and on larger specimen scales beyond our investigations. We calculated the relation between velocity variation of the full-length ultrasonic signals for both uniaxial compression and uniaxial tension using a linear regression for elastic material behavior up to 30% of the respective stress which agrees with previous studies [4,6,27]. Furthermore, we evaluated shrinkage and creep processes equally by means of dv/v and CC to indicate long-term changes to the ultrasonic signals. These long-term changes must be considered for signal comparisons over time. Calibration of the effect of mechanical loads adds to the list of all conceivable impacts on concrete structures such as temperature or moisture, and must be quantifiable to separate reversible from irreversible changes, i.e., damage. With our research, we contribute to characterizing the impact of mechanical loads on ultrasound propagating in concrete.

## Figures and Tables

**Table 1:** Concrete raw materials and their composition for all experiments in this study, where the aggregates are primarily quartzitic and crushed.

Cement Content	Water/Cement	Aggregate Size			Superplasticizer
CEM I 42.5 R	w/c	0/2 mm	2/5 mm	5/8 mm	BASF MasterEase 3880
350 kg/m³	0.45	46.5%	34.0%	19.5% *	2.5% **

* Contains 3.0% oversized grains 8/12 mm. ** As a mass fraction in % of the cement content.

## Data Availability

This research was conducted with respect to the Berlin Declaration on Open Access to Knowledge in the Sciences and Humanities. The research unit *Concrete Damage Assessment by Coda Waves (CoDA)* provides the data and methods using a mySQL-based database. Access may be queried by visiting www.mae.ed.tum.de/coda, accessed on 1 June 2022.
